# Haptoglobin Phenotype, Preeclampsia Risk and the Efficacy of Vitamin C and E Supplementation to Prevent Preeclampsia in a Racially Diverse Population

**DOI:** 10.1371/journal.pone.0060479

**Published:** 2013-04-03

**Authors:** Tracey L. Weissgerber, Robin E. Gandley, Paula L. McGee, Catherine Y. Spong, Leslie Myatt, Kenneth J. Leveno, John M. Thorp, Brian M. Mercer, Alan M. Peaceman, Susan M. Ramin, Marshall W. Carpenter, Philip Samuels, Anthony Sciscione, Margaret Harper, Jorge E. Tolosa, George Saade, Yoram Sorokin, for the Eunice Kennedy Shriver National Institute of Child Health and Human Development Maternal-Fetal Medicine Units Network

**Affiliations:** 1 Division of Nephrology and Hypertension, Mayo Clinic, Rochester, Minnesota, United States of America; 2 Department of Obstetrics, Gynecology and Reproductive Science, University of Pittsburgh, Pittsburgh, Pennsylvania, United States of America; 3 The George Washington University Biostatistics Center, Washington, DC, United States of America; 4 The Eunice Kennedy Shriver National Institute of Child Health and Human Development, Bethesda, Maryland, United States of America; Department of Obstetrics and Gynecology of University of Cincinnati, Cincinnati, Ohio, United States of America; 6 University of Texas Southwestern Medical Center, Dallas, Texas, United States of America; 7 University of North Carolina at Chapel Hill, Chapel Hill, North Carolina, United States of America; 8 Case Western Reserve University-MetroHealth Medical Center, Cleveland, Ohio, United States of America; 9 Northwestern University, Chicago, Illinois, United States of America; 10 University of Texas Health Science Center at Houston-Children’s Memorial Hermann Hospital, Houston, Texas, United States of America; 11 Brown University, Providence, Rhode Island, United States of America; 12 The Ohio State University, Columbus, Ohio, United States of America; 13 Drexel University, Philadelphia, Pennsylvania, United States of America; 14 Wake Forest University Health Sciences, Winston-Salem, North Carolina, United States of America; 15 Oregon Health and Science University, Portland, Oregon, United States of America; 16 University of Texas Medical Branch, Galveston, Texas, United States of America; 17 Wayne State University, Detroit, Michigan, United States of America; VU University Medical Center, The Netherlands

## Abstract

Haptoglobin’s (Hp) antioxidant and pro-angiogenic properties differ between the 1-1, 2-1, and 2-2 phenotypes. Hp phenotype affects cardiovascular disease risk and treatment response to antioxidant vitamins in some non-pregnant populations. We previously demonstrated that preeclampsia risk was doubled in white Hp 2-1 women, compared to Hp 1-1 women. Our objectives were to determine whether we could reproduce this finding in a larger cohort, and to determine whether Hp phenotype influences lack of efficacy of antioxidant vitamins in preventing preeclampsia and serious complications of pregnancy-associated hypertension (PAH). This is a secondary analysis of a randomized controlled trial in which 10,154 low-risk women received daily vitamin C and E, or placebo, from 9-16 weeks gestation until delivery. Hp phenotype was determined in the study prediction cohort (n = 2,393) and a case-control cohort (703 cases, 1,406 controls). The primary outcome was severe PAH, or mild or severe PAH with elevated liver enzymes, elevated serum creatinine, thrombocytopenia, eclampsia, fetal growth restriction, medically indicated preterm birth or perinatal death. Preeclampsia was a secondary outcome. Odds ratios were estimated by logistic regression. Sampling weights were used to reduce bias from an overrepresentation of women with preeclampsia or the primary outcome. There was no relationship between Hp phenotype and the primary outcome or preeclampsia in Hispanic, white/other or black women. Vitamin supplementation did not reduce the risk of the primary outcome or preeclampsia in women of any phenotype. Supplementation increased preeclampsia risk (odds ratio 3.30; 95% confidence interval 1.61–6.82, p<0.01) in Hispanic Hp 2-2 women. Hp phenotype does not influence preeclampsia risk, or identify a subset of women who may benefit from vitamin C and E supplementation to prevent preeclampsia.

## Introduction

Preeclampsia affects 2–7% of pregnancies [1], [2]. Diagnosed as new onset hypertension and proteinuria after 20 weeks gestation [1], [2], preeclampsia is a leading cause of maternal [3] and fetal [4] morbidity and mortality. Oxidative stress (including low antioxidant levels) and angiogenic imbalance are believed to contribute to preeclampsia [5], [6], [7]. No intervention has been proven to prevent preeclampsia. Vitamin C and E lowered preeclampsia incidence by 60% in a pilot randomized controlled trial (RCT) in high-risk women [8]. Unfortunately, this effect was not confirmed in subsequent RCTs involving high [9], [10], [11], [12], [13] or low-risk women [13], [14], [15]. Although the discordant effects in large and small populations are likely due to reduced power in small studies, differences might also be explained by increased heterogeneity in large multicenter studies obscuring a responsive subset of women.

**Figure 1:**
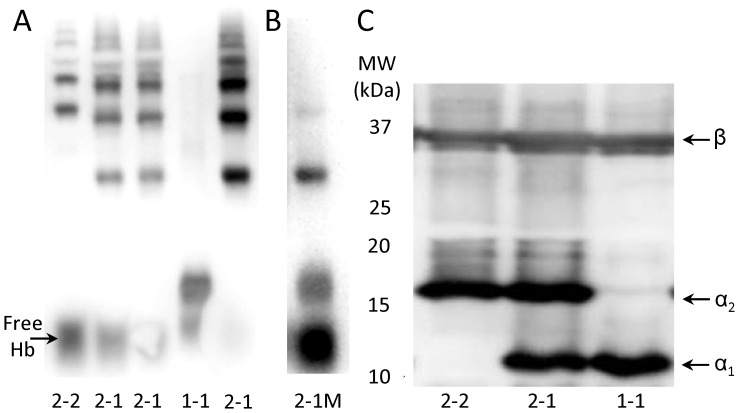
Hp phenotypes by Native and SDS PAGE. Panel A: Hp phenotyping of Hb-supplemented serum by Native PAGE on a 6% gel. Hp 2-2 (Lane 1) is a heterodimer with a series of slow moving bands. Hp 1-1 (Lane 4) is a homodimer with one fast moving band. Hp 2-1 (Lanes 2, 3, 5) has a band between the Hb/Hp 1-1 and Hb/Hp 2-2 bands, and several slow-moving bands in the same region as the Hb/Hp 2-2 bands. Panel B: In the rare Hp 2-1 M phenotype, a modification of the Hp α_2_ allele leads to overproduction of the fast migrating Hp 1-1 band, relative to the slower migrating Hp 2-1 bands. This results in a stronger Hb/Hp 1-1 band, and weaker Hb/Hp 2-1 bands, than in Hp 2-1. Panel C: Hp phenotyping of serum by SDS PAGE on a 12% gel. The Hp β band is common to all phenotyes. The presence of the Hp 1 and 2 alleles is indicated by the α_1_ and α_2_ bands, respectively.

The phenotype of the antioxidant [16] and pro-angiogenic [17] protein haptoglobin (Hp) predicts cardiovascular disease risk, and responsiveness to Vitamin E [18], [19], [20] or C and E [21] in some non-pregnant populations. We examined whether Hp phenotype might influence preeclampsia risk, or identify a subset of women who responded to vitamin supplementation. Hp stimulates angiogenesis [17] and acts as a powerful antioxidant by clearing free hemoglobin following hemolysis [16]. Hp’s two different α alleles (α_1_, α_2_) give rise to three genetically determined phenotypes (1-1, 2-1 and 2-2) [16]. Size and structural differences (Figure 1) lead to functional differences between phenotypes [16]. Hp 1-1 is a stronger antioxidant [16], whereas Hp 2-2 is more angiogenic [17]. Less is known about the function of Hp 2-1, a highly variable heterodimer which is not commercially available. Hp 2-1 individuals have no Hp 2-2, very little Hp 1-1, and large amounts of the 2-1 forms which are structurally very different from Hp 1-1 or 2-2 [16].

We chose to examine Hp phenotype and preeclampsia for three reasons. First, Hp’s antioxidant and pro-angiogenic properties depend on phenotype [17], [22], [23]. Oxidative stress and reduced angiogenesis contribute to preeclampsia [5], [6]. In our recent case-control study, Hp 2-1 was associated with increased preeclampsia risk in non-hispanic white women, but not in black women [24]. We sought to confirm these findings in a larger cohort while extending the existing literature to include Hispanics. Second, any potential effect of Hp phenotype on preeclampsia risk or treatment response would affect a significant number of women. Hp 1-1, 2-1 and 2-2 are all common in whites, blacks and Hispanics (prevalences of 16%-50%) [25]. Third, Hp phenotype predicts cardiovascular disease risk, and responsiveness to Vitamin E [18], [19], [20] or C and E [21] in other non-pregnant populations. Hp 2-2 diabetics are twice as likely to have a cardiovascular event as Hp 1-1 and 2-1 diabetics [18], [21], [26]. Vitamin E supplementation eliminates this increased risk in Hp 2-2 diabetics, but has no effect in Hp 1-1 or 2-1 diabetics [18], [19], [27]. In contrast, Vitamin C may be harmful in Hp 2-2 diabetics as oxidation of Vitamin C may exacerbate pre-existing oxidative stress [28]. In post-menopausal women with coronary artery disease, Vitamin C and E decreased coronary artery diameter in Hp 2-2 diabetic women [21]. This harmful effect was not universal, as Vitamin C and E benefited Hp 1-1 women by significantly increasing coronary artery diameter [21].

We sought to determine whether Hp phenotype was associated with serious complications of pregnancy-associated hypertension (PAH) or preeclampsia in white, black or Hispanic women. We also examined whether Hp phenotype might influence responsiveness to antioxidant vitamins in preventing preeclampsia. Based on our previous findings, we hypothesized that Hp 2-1 would be associated with increased preeclampsia risk in white women, but not in black women. We also posited that phenotype would affect treatment response although directionality was difficult to predict based upon data from non-pregnant populations.

## Materials and Methods

### Population

This was a secondary analysis of a *Eunice Kennedy Shriver* National Institute of Child Health and Human Development Maternal-Fetal Medicine Units Network double-blind, multicenter RCT (NCT 00135707) in which 10,154 nulliparous, low risk women received daily doses of 400 IU of vitamin E and 1000 mg of vitamin C, or placebo, from 9-16 weeks gestation until delivery. The trial was conducted at 16 centers in the US between 2003 and 2008. Full details of the study were reported previously [14]. The trial was conducted in accordance with the Declaration of Helsinki of the World Medical Association. All subjects provided written, informed consent before participating. Hp phenotyping was performed at the University of Pittsburgh (Institutional Review Board approval PRO10010368).

### Outcomes

The primary outcome was severe PAH, or mild or severe PAH with elevated liver enzymes (aspartate aminotransferase ≥100 U per liter), elevated serum creatinine (≥1.5 mg/dl), thrombocytopenia (<100,000 platelets/mm^3^), eclampsia, fetal growth restriction (<3^rd^ centile adjusted for sex and race/ethnicity), medically indicated preterm birth before 32 weeks due to PAH, or fetal death after 20 weeks gestation, or neonatal death. Hypertension was diagnosed using blood pressure measurements obtained during or after 20 weeks gestation, excluding intraoperative and intrapartum systolic blood pressures. Severe PAH was defined as a systolic blood pressure of 160 mm Hg or more, or a diastolic blood pressure of 110 mm Hg or more on two occasions 2 to 240 hours apart, or a single severely elevated blood pressure measurement that led to treatment with antihypertensive medication. Mild PAH was defined as systolic pressure between 140 and 159 mm Hg or a diastolic pressure between 90 mm Hg and 109 mm Hg on two occasions 2 to 240 hours apart. Mild preeclampsia was defined as mild PAH with proteinuria documented within the 3 days preceding or following the elevated blood pressure measurement. Proteinuria was defined as protein excretion ≥300 mg/24 hours, a dipstick urinary protein ≥2+, or protein:creatinine ≥0.35 in the absence of a 24-hour urine sample. Early onset preeclampsia was defined as preeclampsia requiring delivery before 37 weeks gestation. Severe preeclampsia was defined as preeclampsia with either severe PAH or protein excretion ^3^5 g/24 hours, or as mild PAH with oliguria (<500 ml urine/24 hours), pulmonary edema (confirmed by radiography), or thrombocytopenia (<100,000 platelets/mm^3^).

### Design

**Figure 2:**
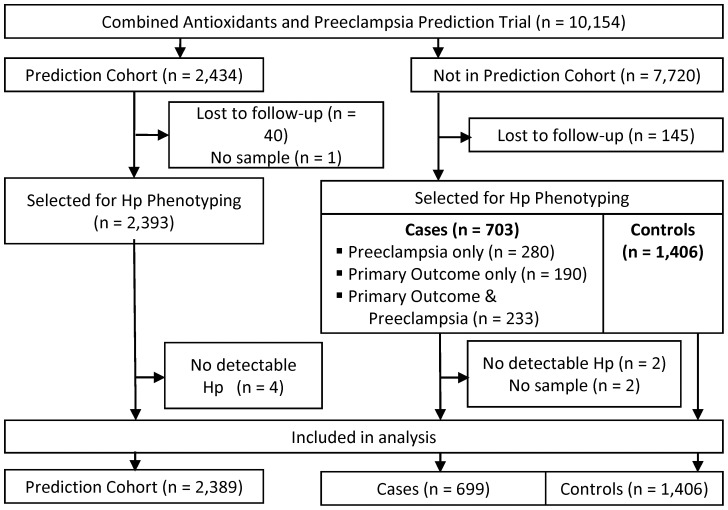
Study Flow Chart. Subjects lost to follow-up include 183 women who miscarried, one subject whose data were removed at the patient’s request, and one subject whose data were removed at the institutional review board’s request.

The RCT included 10,154 women, of whom 2,434 were enrolled in a nested prediction cohort. The RCT design specified that secondary analyses requiring blood be conducted in the prediction cohort, as these women consented to extra blood collection for prediction and pathophysiology studies. If additional subjects were required, investigators could obtain samples from the 7,720 women not in the prediction cohort, in whom very little blood was collected. We needed samples from all white, black, and Hispanic women with preeclampsia in the RCT to detect a 2-fold increase in preeclampsia risk in Hp 2-1, compared to Hp 1-1, in each racial group (α = 0.05; Power: 83% white, 96% black, 98% Hispanic). We determined Hp phenotype in the prediction cohort; then added a case-control cohort composed of women who were not in the prediction cohort. The case-control cohort allowed us to capture all cases without phenotyping 5,500 additional controls. Cases in the case-control cohort included all women with preeclampsia, the primary outcome, or both (n = 703 cases) who were not in the prediction cohort. We selected 2 controls per case (n = 1,406) [29], matched for center and race/ethnicity (Figure 2).

### Hp Phenotyping

Hp phenotyping was performed as described previously [24], [30]. Serum samples (5 µl) supplemented with human hemoglobin (Sigma-Aldrich, St. Louis, MO) were phenotyped by hemoglobin-Hp complex peroxidase activity on native polyacrylamide gel electrophoresis (PAGE). Hemolyzed samples and samples with low Hp concentrations were phenotyped by Western blot following sodium dodecyl sulfate (SDS) PAGE of 1 to 6 µl of serum. Proteins from native and SDS gels were transferred to a polyvinylidene fluoride (PVDF) membrane (Millipore, Billerica, MA). SDS PAGE was followed by Western blotting with primary (1∶5,000, Polyclonal Rabbit Anti-Human Haptoglobin, DakoCytomation, Carpinteria, CA) and secondary (1∶25,000, Goat anti-Rabbit IgG horseradish peroxidase, Millipore) antibodies. Membranes were stained for peroxidase activity (SuperSignal West Pico Chemiluminescent Substrate, Fisher Scientific, Pittsburgh, PA) and imaged (FlouroChem Q System, Cell Biosciences, Santa Clara, CA). Hp phenotypes were identified by banding patterns of the Hp-hemoglobin complexes (native PAGE, Figure 1) or the Hp α_1_ and α_2_ alleles (SDS PAGE).

### Plasma Ascorbate

Blood was collected in vials containing metaphosphoric acid, and frozen at −80 C [31]. Plasma ascorbic acid was measured by high performance liquid chromatography in the Molecular Epidemiology and Biomarker Laboratory (University of Minnesota) as described previously [31].

### Statistical Analysis

The prediction and case-control cohorts were analyzed separately (statistical methods in Data S1); then pooled to maximize power. In the pooled analysis, sampling weights were applied to reduce potential bias arising from an overrepresentation of women with the primary outcome (but not preeclampsia) in the control group for preeclampsia analyses, and an oversampling of women with preeclampsia (but not the primary outcome) in the control group for primary outcome analyses. Patients from the prediction cohort were assigned a weight of one. Cases and controls were assigned a sample weight defined as the inverse of their probability of selection [32]. Sample weights included a post-stratification adjustment for race/ethnicity. Baseline characteristics were compared using the t-test for continuous variables and the Rao-Scott chi-square test [33] for categorical variables. Logistic regression models were constructed to assess the multivariable association between Hp phenotype and the incidence of each outcome, adjusting for variables that were associated with Hp phenotype in univariate analyses. Odds ratios and 95% confidence intervals were estimated from unconditional survey logistic regression models. Taylor series replicate weights were created to estimate standard errors and p values in the weighted analyses. Model covariates included randomization group, and demographic variables that differed between women with the Hp 1-1, 2-1 and 2-2 phenotypes (age, self-reported vitamin use, education level, diastolic blood pressure at screening). The weighted analysis gave similar results to an unweighted analysis (i.e. samples were not weighted by the post-stratification sample weights; data not shown). Results were considered to be statistically significant at α = 0.05 with no adjustment for multiple comparisons [34]. All p values are 2-sided. SAS Version 9.2 (SAS Institute Inc.) and STATA (StataCorp, 2011 *Stata Statistical Software: Release 12*, College Station, TX, StataCorp LP), using procedures appropriate for sample-weighted data, were used for all analyses.

## Results

### Cohorts

We phenotyped 2,393 of the 2,434 women in the prediction cohort (Figure 2), excluding 40 women who miscarried, and one who had no samples collected. The case-control cohort included all remaining subjects who developed the primary outcome only (severe PAH, or mild or severe PAH with elevated liver enzymes, elevated serum creatinine, thrombocytopenia, eclampsia, fetal growth restriction, medically indicated preterm birth or perinatal death) (n = 190), preeclampsia only (n = 280), or the primary outcome and preeclampsia (n = 233), and 1,406 controls matched for center and race/ethnicity. Women in the prediction cohort entered the study at an earlier gestational age (11.4±1.1) than women in the case-control cohort (14.0±2.0). Results of separate analyses for the prediction (Data S1; Tables S1, S2) and case-control cohorts (Data S1; Tables S3, S4, S5, S6, S7) are presented in Data S1. Results for the pooled cohort are described below.

### Subjects

The pooled dataset included 1,008 Hp 1-1, 2,129 Hp 2-1 and 1,261 Hp 2-2 women. Ninety-six women had the rare 2-1 M phenotype, which results from a modification of the α_2_ allele [16]. These women were excluded from subsequent analyses. There were 1,840 white women, 1,392 black women, 1,188 Hispanic women, and 74 women of “Other” race.

### Weighting

The weighted sample included 2,201 Hp 1-1 women, 4,840 Hp 2-1 women, 2,760 Hp 2-2 women, and 146 Hp 2-1 M women. The weighted cohort accurately reproduced the demographic characteristics of the original sample (Table S8). Demographic characteristics in the weighted cohort were not significantly different between the placebo and vitamin groups.

### Subject Characteristics

As expected, race/ethnicity differed between women with the Hp 1-1, 2-1, 2-2 and 2-1 M phenotypes (Table 1). The phenotype distribution for each race/ethnicity was similar to previous reports (Table 1, S9) [25]. Hp phenotype groups did not differ with respect to pre-pregnancy BMI, blood pressure at randomization, smoking, history of a previous pregnancy, or family history of preeclampsia (Table 1). Subsequent analyses were adjusted for characteristics that differed between the Hp 1-1, 2-1 and 2-2 groups (race/ethnicity, age, education, vitamin use). Analyses were also adjusted for diastolic blood pressure at randomization, which differed between phenotype groups in the prediction cohort (Table S10) but not in the pooled cohort. The white and other groups were pooled for race/ethnicity adjustments, as phenotype prevalence in the other group was most similar to whites (Table S9).

**Table 1:** Subject Characteristics for the Weighted Pooled Cohort by Hp phenotype.

Subject Characteristics	Hp 1-1 (n = 2,201)	Hp 2-1 (n = 4,840)	Hp 2-2 (n = 2,760)	Hp 2-1 M (n = 161)	p
Age – years	22.4±4.9	23.6±5.1*	24.0±5.1*	20.2±3.7*^,†,‡^	<0.01
Gestational age at randomization – week	13.4±2.1	13.3±2.0	13.4±2.1	13.0±2.5	0.48
Race or ethnicity - % within phenotype					NA
White	618 (28%)	2,142 (44%)	1,408 (51%)	3 (2%)	
Black	779 (35%)	1,059 (22%)	542 (20%)	132 (82%)	
Hispanic	779 (35%)	1,555 (32%)	724 (26%)	25 (6%)	
Other	25 (1%)	84 (2%)	86 (3%)	1 (<1%)	
Pre-pregnancy body mass index - kg/m^2^	25.3±6.1	24.9±5.4	25.0±5.6	26.6±8.7	0.20
Smoked during pregnancy – n (%)	352 (16%)	721 (15%)	515 (19%)	23 (15%)	0.12
Education - years	12.2±2.9	12.7±2.7*	13.1±2.6*^,†^	12.0±2.0^†,‡^	<0.01
Prenatal/multivitamin use prior to randomization – n (%)	1,517 (69%)	3,759 (78%)*	2,194 (79%)*	132 (82%)*	<0.01
Previous pregnancy – n (%)	520 (24%)	1,084 (22%)	731 (26%)	33 (21%)	0.16
Family history of preeclampsia – n (%)	319 (14%)	634 (13%)	341 (12%)	18 (11%)	0.64
Blood pressure at entry (9-12 weeks)					
Systolic – mm Hg	108±10	108±10	108±10	110±9	0.23
Diastolic – mm Hg	65±8	65±8	65±8	64±9	0.25

Values are mean ± SD or n (%). Abbreviations: NA; not applicable. Statistical comparisons for race/ethnicity were not performed, as cases and controls were matched for race/ethnicity.

Significant difference (p<0.05) from: *Hp 1-1, ^†^Hp 2-1, ^‡^Hp 2-2.

### Primary Outcome and Preeclampsia Risk

Hp phenotype was not significantly associated with the risk of the primary outcome, preeclampsia, severe preeclampsia, or early onset preeclampsia in white/other, Hispanic or black women (Table 2). Women of “other” race comprised 4% of the white/other group, and results were not different when these women were excluded (data not shown). The relationship between Hp phenotype on early onset preeclampsia approached statistical significance (p = 0.06) in Hispanics.

**Table 2:** Outcomes according to race/ethnicity and Hp phenotype in the weighted pooled cohort.

Outcome	Weighted Cases (n)	Hp 2-1† OR (95% CI)	Hp 2-2† OR (95% CI)
White/Other			
Primary Outcome*	216	0.87 (0.56, 1.35)	0.92 (0.58, 1.45)
Preeclampsia	222	0.98 (0.62, 1.54)	1.18 (0.74, 1.88)
Severe Preeclampsia	90	1.30 (0.65, 2.62)	1.31 (0.64, 2.69)
Early Onset Preeclampsia	58	0.96 (0.41, 2.22)	1.40 (0.61, 3.22)
Late Onset Preeclampsia	163	0.98 (0.59, 1.64)	1.10 (0.65, 1.87)
Black			
Primary Outcome	243	1.26 (0.90, 1.76)	1.00 (0.66, 1.51)
Preeclampsia	237	1.03 (0.74, 1.45)	0.92 (0.61, 1.38)
Severe Preeclampsia	115	1.14 (0.73, 1.80)	0.88 (0.49, 1.55)
Early Onset Preeclampsia	74	1.18 (0.67, 2.07)	0.95 (0.48, 1.88)
Late Onset Preeclampsia	163	0.97 (0.66, 1.44)	0.90 (0.56, 1.46)
Hispanic			
Primary Outcome	116	0.79 (0.49, 1.29)	0.85 (0.47, 1.54)
Preeclampsia	217	0.77 (0.53, 1.12)	0.74 (0.46, 1.19)
Severe Preeclampsia	76	0.71 (0.40, 1.24)	0.67 (0.32, 1.40)
Early Onset Preeclampsia	42	0.41 (0.20, 0.86)	0.53 (0.21, 1.37)
Late Onset Preeclampsia	175	0.90 (0.60, 1.37)	0.82 (0.49, 1.39)

Hp 1-1 was the reference group for odds ratios. Hp phenotype was determined in 1,573 black women, 1,164 Hispanic women, and 1,432 women of white or other race. Each observation was weighted to reflect the pregnancy outcomes and racial distribution of the full cohort.

* The primary outcome was severe PAH, or mild or severe PAH with elevated liver enzymes, elevated serum creatinine, thrombocytopenia, eclampsia, fetal growth restriction, medically indicated preterm birth or perinatal death.

Abbreviations: OR, odds ratio; CI, confidence interval.

† Adjusted for group (treatment vs. placebo), race/ethnicity, age, education, vitamin use and diastolic blood pressure at randomization.

### Treatment Response

Vitamin C and E supplementation did not prevent the primary outcome or any form of preeclampsia among white/other, black or Hispanic women of any Hp phenotype after adjusting for vitamin use, age, education, and diastolic blood pressure at randomization (data not shown).

In Hispanics, the effect of vitamin supplementation differed between Hp phenotype groups for preeclampsia (Hp phenotype*treatment group: p<0.01) and late onset preeclampsia (p = 0.02), but not for severe (p = 0.06) or early onset preeclampsia (p = 0.10). Vitamin supplementation significantly increased preeclampsia (odds ratio (OR) 3.30; 95% confidence interval (CI) 1.61-6.82, p<0.01, Table 3) and late onset preeclampsia risk (3.01; 1.38-6.57, p<0.01) in Hispanic Hp 2-2 women. This adverse effect was not seen in Hp 1-1 or 2-1 Hispanics (p>0.05), or in white/other or black women of any phenotype.

**Table 3:** Odds ratios for effect of treatment in Hispanic women in the weighted pooled cohort.

Outcome	Phenotype	OR (95% CI)*	p*
Preeclampsia	Hp 1-1	0.71 (0.39, 1.29)	0.27
	Hp 2-1	0.95 (0.61, 1.50)	0.83
	Hp 2-2	3.30 (1.61, 6.82)	<0.01
Late Onset Preeclampsia	Hp 1-1	0.70 (0.36, 1.35)	0.29
	Hp 2-1	1.02 (0.63, 1.66)	0.92
	Hp 2-2	3.01 (1.38, 6.57)	<0.01

* Adjusted for vitamin use, age, education and diastolic blood pressure at randomization.

Increased preeclampsia risk in Hispanic Hp 2-2 women was specific to the large quantities of vitamins C and E in study supplements. Prenatal/multivitamin use at randomization was not associated with an increased risk of preeclampsia (2.17; 0.53-8.71, p = 0.27) or late onset preeclampsia (2.85; 0.57–14.36, p = 0.20) in Hispanic Hp 2-2 women receiving placebo. Low rates of prenatal/multivitamin use at randomization (43% in Hispanics vs. 91% in non-Hispanics, Table S10) are unlikely to explain why the adverse effect of supplementation in Hp 2-2 women was only seen in Hispanics. Hispanic 2-2 women who were not taking prenatal vitamins at randomization had a smaller increase in preeclampsia risk with supplementation than Hispanic 2-2 women who were taking prenatal vitamins at randomization (2.01; 0.80–5.01, p = 0.14 vs. 7.83; 2.01–30.53, p<0.01, Table 4).

**Table 4:** Odds ratios for treatment effect in Hispanic Hp 2-2 women according to prenatal/multivitamin use at randomization.

Outcome	Vitamin Use at Randomization	OR (95% CI)*	p*
Preeclampsia	Yes	7.83 (2.01, 30.53)	<0.01
	No	2.01 (0.80, 5.01)	0.14
Late Onset Preeclampsia	Yes	8.23 (1.64, 41.28)	0.01
	No	1.91 (0.73, 5.00)	0.18

* Adjusted for age, education and diastolic blood pressure at randomization.

### Hp Phenotype, Prenatal/Multivitamin Use at Randomization, and Plasma Ascorbate

Hp phenotype influences plasma ascorbate concentration [35], providing a potential mechanism by which Hp phenotype might influence treatment response to vitamin C and E. We examined the relationship between Hp phenotype and plasma ascorbate in a pre-planned analysis. Plasma ascorbate was measured at the pre-randomization screening visit in 264 women with known Hp phenotypes. Plasma ascorbate was lower in women who were not taking a prenatal/multivitamin at randomization (n = 34) compared to those who were (n = 230) (p = 0.02 for main effect, Table 5). Statistical comparisons within phenotype groups were not conducted, as the number of women who were not taking prenatal/multivitamins at randomization within each phenotype group was very small. Hp phenotype was not significantly associated with plasma ascorbate (p = 0.73). The interaction between Hp phenotype and vitamin use at randomization was not significant (p = 0.32). Plasma ascorbate was only measured in six Hispanic Hp 2-2 women; therefore we could not determine whether supplementation had a different effect on plasma ascorbate in this group.

**Table 5:** Plasma ascorbate concentrations according to Hp phenotype and prenatal/multivitamin use at randomization.

Hp Phenotype	Not Taking Prenatal/multivitaminsat Randomization	Taking Prenatal/multivitaminsat Randomization
All	8.4 (7.2, 9.5) (n = 34)	9.9 (9.4, 10.3) (n = 230)*
1-1	9.3 (7.4, 11.3) (n = 12)	9.6 (8.6, 10.5) (n = 48)
2-1	8.0 (6.3, 9.6) (n = 16)	9.8 (9.2, 10.4) (n = 126)
2-2	7.4 (4.7, 10.1) (n = 6)	10.2 (9.3, 11.1) (n = 56)

Plasma ascorbate at the pre-randomization study visit was measured in a subset of 264 patients with known Hp phenotype. The vitamin and placebo groups were pooled, as study treatment had not yet started. Women not taking a prenatal vitamin at randomization had lower plasma ascorbate than women taking prenatal/multivitamins at randomization (*p = 0.02). Statistical comparisons within phenotype group were not conducted due to the small number of women not taking prenatal/multivitamins at randomization. Values are mean (95% confidence interval) in mg/L.

## Discussion

Hp phenotype was not associated with the risk of the primary outcome (a composite of PAH and serious adverse outcomes in the mother, her fetus or neonate) or any type of preeclampsia in white, black or Hispanic women. Moreover, daily vitamin C and E supplementation did not prevent the primary outcome or preeclampsia in women of any Hp phenotype. Supplementation increased the risk of preeclampsia and late onset preeclampsia by 3-fold in Hispanic Hp 2-2 women.

### Hp Phenotype and the Risk of Preeclampsia and Serious Complications of PAH

We found no relationship between Hp phenotype and the primary outcome or preeclampsia risk in white, black or Hispanic women. This study has two key strengths. First, it extends the existing literature by including large numbers of black and Hispanic women. Previous studies focused on predominantly white, non-Hispanic women [24],[36],[37],[38]. Studies examining the relationship between Hp phenotype and preeclampsia risk in different racial groups were recently recommended [39]. Second, this is the largest study of non-Hispanic white women completed to date, and was adequately powered to detect the difference that we observed in our previous case-control study [24]. This is particularly important, as the heterogeneous results and small sample sizes of previous European studies suggests that spurious findings may be common. At the time we initiated this investigation, two small, underpowered European studies had reported that preeclampsia risk was not different [38] or increased [36] in Hp 1-1 white women. Adequately powered studies [39], and studies in North American women, were needed. An Israeli study subsequently reported a 50% reduction in preeclampsia risk among Hp 1-1 women, compared to Hp 2-1 and 2-2 [24], [30], [37]. Our larger case-control study suggested that Hp 1-1 was only protective when compared to Hp 2-1, and not Hp 2-2 [24]. Unfortunately, we were unable to confirm this finding in the present cohort. However, we did confirm our previous preliminary data [24] suggesting that there was no relationship between Hp phenotype and preeclampsia risk in black women in a large adequately powered cohort. The trend (p = 0.06) towards an increased risk of early onset preeclampsia in Hp 2-1 and 2-2 Hispanics was likely a spurious result due to the small number of cases (n = 42).

### Hp Phenotype and Vitamin C and E

Accumulating evidence suggests that preeclampsia and later life cardiovascular disease are intimately linked. These conditions share several risk factors (i.e. black race, obesity, diabetes) and pathophysiological processes (inflammation, oxidative stress, endothelial dysfunction, and impaired angiogenesis) [40], [41]. The American Heart Association recently recognized preeclampsia as a cardiovascular disease risk factor [41]. Hp phenotype affects cardiovascular disease risk and treatment response to antioxidant vitamins in some non-pregnant populations, particularly in diabetics [18], [19], [21], [26]. Uncomplicated pregnancy is an insulin resistant state [42], and gestational diabetes [43] and Type 1 diabetes [44] increase preeclampsia risk. Therefore, we examined the potential for Hp phenotype to influence the effectiveness of vitamin C and E in preventing preeclampsia and serious complications of PAH.

Daily vitamin C and E did not prevent preeclampsia or serious complications of PAH in low risk primiparous women of any Hp phenotype. Supplementation actually increased preeclampsia risk by 3-fold in Hispanic Hp 2-2 women, however the mechanism requires further investigation. This effect was not explained by the strikingly lower prevalence of vitamin supplementation at randomization in Hispanics. Hispanic women have an increased risk of the metabolic syndrome and diabetes, conditions associated with oxidative stress [45]. Platelet volume, a marker of accelerated platelet turn over, was increased in Hispanic women compared to white or black women in the prediction cohort [46]. Accelerated platelet turnover could indicate low-grade intravascular coagulation and subsequent mild chronic hemolysis. The resulting oxidative stress would be worse in Hp 2-2 women due to the low antioxidant capacity of this phenotype. We speculate that supplementation may have exacerbated pre-existing oxidative stress, and therefore preeclampsia risk, in Hispanic Hp 2-2 women by increasing ascorbate radical formation. A definitive explanation will be sought in future studies. Supplementation increased the risk of late onset preeclampsia, but not early onset preeclampsia, in Hp 2-2 Hispanics. This could reflect pathophysiological differences between these conditions or lower power due to fewer cases of early onset preeclampsia.

Several factors could explain the difference between our results and previous research in other populations [18], [19], [21], [26]. First, cardiovascular disease studies suggest that vitamin E alone has different effects from vitamin C and E combined [18], [19], [27]. Vitamin C and E can be beneficial or harmful, depending on Hp phenotype [27]. Coronary artery diameter increased following vitamin C and E supplementation in Hp 1-1 post-menopausal women with coronary artery disease. However, the pro-oxidant effects of vitamin C [28] combined with the reduced antioxidant capacity of Hp 2-2 may have contributed to accelerated coronary artery narrowing in diabetic Hp 2-2 post-menopausal women taking vitamins C and E [21]. Oxidative stress increases in pregnancy [40], suggesting that a similar mechanism could be active in Hp 2-2 pregnant women. In partial support of this hypothesis, we observed a 3-fold increase in preeclampsia risk in Hp 2-2 Hispanic women receiving vitamin C and E. The original RCT combined vitamins C and E, an approach used in cardiovascular disease prevention trials [47], [48], [49] that was successful in small clinical trials. Therefore, we cannot determine whether vitamin E or C alone would have been efficacious. However, the absence of a beneficial treatment effect in women of any Hp phenotype makes this possibility unlikely.

Second, previous studies focused primarily on Type 2 diabetics [18], [19], [21], [26], however women with pre-existing diabetes were excluded from the current study. Insulin resistance increases during pregnancy, and 3% of women in the trial developed gestational diabetes [42]. The most extensively studied mechanism by which vitamin E reduces cardiovascular disease risk in Type 2 diabetes is by preventing the HDL dysfunction caused by cross-linking with glycosylated hemoglobin [28]. Hypothesizing that a similar mechanism might be active in Type 1 diabetes, members of our study team assessed the potential for Hp phenotype to modify the effectiveness of vitamin C and E in preventing preeclampsia in women with Type 1 diabetes [30]. Unfortunately, supplementation was not effective in preventing preeclampsia in women with Type 1 diabetes of any Hp phenotype [30]. Trials in Type 2 diabetics have not been conducted.

Third, despite the similarities between preeclampsia and cardiovascular disease, there are also critical differences. Preeclampsia only occurs in pregnancy, and is proposed to result from an interaction between materials released by the placenta, and maternal constitutional characteristics that are largely cardiovascular risk factors [40]. It may be that transient exposure to insults in young women cannot be equated to years of chronic exposure to cardiovascular risk factors in older women.

### Conclusions

In contrast to our hypothesis, our secondary analysis of a large RCT indicates that Hp phenotype does not influence preeclampsia risk, or identify a subset of nulligravidas for whom vitamins C and E would reduce preeclampsia risk in white, black or Hispanic women. Vitamins C and E supplementation may increase preeclampsia risk in Hispanic Hp 2-2 women, but due to the limitations of secondary analysis, this finding requires verification.

## Supporting Information

Data S1 **Supplemental Data.** Supplemental data file containing methods and results for separate analyses of prediction cohort and case-control cohort, and an appendix listing members of the *Eunice Kennedy Shriver* National Institute of Child Health and Human Development Maternal-Fetal Medicine Units Network.
(DOC)

Table S1 **Prediction cohort subject characteristics.** Values are mean ± SD or n (% within phenotype). Significant difference (p<0.05) from: *Hp 1-1, ^†^Hp 2-1, ^‡^Hp 2-2.
(DOC)

Table S2 **Outcomes according to Hp phenotype in the prediction cohort.** *Adjusted for treatment group (vitamins vs. placebo), age, race/ethnicity, education, vitamin use prior to randomization, and diastolic blood pressure at randomization. ^†^The primary outcome was severe PAH, or mild or severe PAH with elevated liver enzymes, elevated serum creatinine, thrombocytopenia, eclamptic seizure, fetal growth restriction, medically indicated preterm birth or perinatal death.
(DOC)

Table S3 **Subject Characteristics for Subjects not in the Prediction Cohort, and in the Weighted Case-Control Cohort.** Values are mean ± SD or n (%).
(DOC)

Table S4 **Case-control cohort weighted subject characteristics.** Values are mean ± SD or n (%). Abbreviations: NA; not applicable. Statistical comparisons for race/ethnicity were not performed, as cases and controls were matched for race/ethnicity. Significant difference (p<0.05) from: *Hp 1-1, ^†^Hp 2-1, ^‡^Hp 2-2.
(DOC)

Table S5 **Outcomes according to Hp phenotype and race/ethnicity in the case-control cohort.** Hp 1-1 was the reference group for odds ratios. Hp phenotype was determined in 769 black women, 579 Hispanic women, and 716 women of white or other race. Each observation was weighted to reflect the pregnancy outcomes and racial distribution of the full cohort, excluding women in the prediction study (3,170 white/other women, 1,839 black women, 2,459 Hispanic women). Abbreviations: OR, odds ratio; CI, confidence interval. *Adjusted for group (treatment vs. placebo), race/ethnicity, age, education, vitamin use and diastolic blood pressure at randomization ^†^Significantly different from 1, p<0.05.
(DOC)

Table S6 **Interaction between Hp phenotype and treatment in case-control cohort.** *Adjusted for vitamin use, age, education and diastolic blood pressure at randomization
(DOC)

Table S7 **Odds ratios for the effect of treatment in Hispanic women in the case-control cohort.** *Adjusted for vitamin use, age, education and diastolic blood pressure at randomization
(DOC)

Table S8 **Subject Characteristics for the Original Study and the Weighted Pooled Cohort Values are mean ± SD or n (%).** *Values for the 9,969 women with known pregnancy outcomes in the original study. Women who were lost to follow-up (n = 183) were excluded. Weights for this analysis were based on race/ethnicity and pregnancy outcome, therefore these women were not eligible to be selected for the pooled cohort analysis.
(DOC)

Table S9 **Hp phenotype prevalence by race in the weighted pooled cohort Values are n (% within race).**
(DOC)

Table S10 **Subject Characteristics for Hispanic vs. non-Hispanic Women in the Weighted Pooled Cohort.** Values are mean ± SD unless otherwise indicated.
(DOC)
