# Supplementary material for: Haptoglobin Phenotype, Preeclampsia Risk and the Efficacy of Vitamin C and E Supplementation to Prevent Preeclampsia in a Racially Diverse Population
Source: PLoS One. 2013 Apr 3;8(4):e60479. doi: 10.1371/journal.pone.0060479 (PMC3616124; doi:10.1371/journal.pone.0060479)
Supplement: Data S1 — Supplemental Data. Supplemental data file containing methods and results for separate analyses of prediction cohort and case-control cohort, and an appendix listing members of the Eunice Kennedy Shriver National Institute of Child Health and Human Development Maternal-Fetal Medicine Units Network. (DOC) [file pone.0060479.s001.doc]

**Methods S1**

**Statistical Analysis**

**Prediction Cohort:** Baseline characteristics were compared using Kruskal-Wallis for continuous variables and Fisher’s exact test for categorical variables. The multiple comparison test after Kruskal-Wallis was used to obtain post-hoc pair-wise comparisons between Hp phenotypes at the point 0.05 level with no adjustments for the multiple comparisons.^29^ Logistic regression models were constructed to assess the multivariable association between Hp phenotype and the incidence of each outcome, adjusting for traditional risk factors and other univariately associated variables.

**Case-Control Cohort:** All statistical analysis were completed as described in the manuscript, however women in the prediction cohort were not included.

**Results S1**

**Prediction Cohort**

**Subjects:** Four of the 2,393 women had no detectable Hp (Hp 0), and were excluded from the analysis. Women with the rare 2-1M phenotype (n=55) were excluded from analyses of PAH risk and treatment response as there were too few subjects to draw conclusions.

**Subject Characteristics:** As expected, race/ethnicity differed between women with the 1-1, 2-1, 2-2 and 2-1M phenotypes (Table S1). Phenotype prevalence in whites, blacks and Hispanics was similar to previously reported values for North Americans.^24^ Age increased progressively across the Hp 2-1M, 1-1, 2-1 and 2-2 phenotype groups. Hp 2-2 women were older and more educated than women with the other phenotypes, and were more likely to report pre-randomization vitamin use than Hp 1-1 women. Although the overall p value for diastolic blood pressure at randomization was 0.05, individual comparisons were not statistically significant. Women with the 1-1, 2-1, 2-2 and 2-1M phenotypes did not differ with respect to pre-pregnancy BMI, systolic blood pressure at randomization, smoking, history of a previous pregnancy, or family history of preeclampsia. Hp 2-1M women were randomized slightly earlier than women with the other phenotypes. Although statistically significant, this 2-3 day difference is unlikely to have any clinical impact. Subsequent analyses were adjusted for characteristics that differed between the 1-1, 2-1 and 2-2 groups (race/ethnicity, age, education, vitamin use, and diastolic blood pressure at randomization). The “White” and “Other” groups were pooled for race/ethnicity adjustments, as the phenotype prevalence in the “Other” group was most similar to whites.

**Risk of the Primary Outcome, Gestational Hypertension and Preeclampsia:** There was no significant interaction between race/ethnicity and Hp phenotype for any type of PAH (data not shown). Hp phenotype did not significantly affect the risk of the primary outcome, gestational hypertension, preeclampsia, severe preeclampsia, early onset preeclampsia, or late onset preeclampsia (Table S2).

**Treatment Response:** Vitamin supplementation was not effective in preventing the RCT primary outcome, gestational hypertension, preeclampsia, severe preeclampsia, early onset preeclampsia, or late onset preeclampsia in women of any Hp phenotype (p>0.05 for the Hp phenotype * treatment group interaction, after adjusting for race/ethnicity, age, education, vitamin use at randomization, and diastolic blood pressure at randomization). The Hp phenotype * treatment interaction p value for early onset preeclampsia approached statistical significance (p=0.06), however power was very low as only 32 subjects developed early onset preeclampsia.

**Case-Control Cohort**

**Subjects:** The cohort included 703 cases (Primary outcome: n=423, Preeclampsia: n=513) and 1406 controls. Four cases were excluded because no sample was available (n=2) or Hp was not detectable (Hp 0, n=2). Women with the Hp 2-1M phenotype (n=41) were excluded from risk and treatment response analyses.

**Weighting:** Table S3 shows subject characteristics for the vitamin and placebo groups in all subjects from the original study who were not in the prediction cohort, and in the weighted case-control cohort used for this analysis. The similarity of values suggests that the weighting effectively reproduced the demographic characteristics of the original sample. Demographic characteristics in the weighted case-control cohort were not significantly between the placebo and vitamin groups.

**Subject Characteristics:** The racial distribution of phenotypes was similar to expected values.^24^ Race/ethnicity, age, education, and pre-randomization vitamin use differed between the phenotype groups (Table S4). Subsequent analyses were adjusted for these characteristics. The “White” and “Other” groups were pooled for subsequent analyses, as the phenotype prevalence in the “Other” group was most similar to whites.

**Primary Outcome and Preeclampsia Risk:** Black women with the Hp 2-1 phenotype were significantly more likely to develop the primary outcome than those with the 1-1 phenotype (Table S5; odds ratio: 1.64, 95% confidence interval: 1.08-2.48, p=0.020). This increased risk was not observed for Hp 2-2. There was no relationship between Hp phenotype and primary outcome risk in the white/other or Hispanic racial groups. Among Hispanic women, Hp 2-2 was associated with a significant reduction in preeclampsia risk (0.56, 0.31-1.00, p=0.049), whereas Hp 2-1 was associated with a significant reduction in early onset preeclampsia risk (0.34, 0.14-0.82, p=0.016), compared to Hp 1-1. There was no relationship between Hp phenotype and preeclampsia risk in the white/other or black racial groups.

**Treatment Response:** There was a significant interaction between Hp phenotype and treatment group for preeclampsia in Hispanic women (Table S6, p=0.012), and for late onset preeclampsia in all women (p=0.048) and Hispanic women (p=0.018). Vitamin C and E significantly increased the risk of preeclampsia (Table S7, OR 4.08; 95% CI 1.59-10.43, p=0.003) and late onset preeclampsia (4.37; 1.54-12.43, p=0.06) in Hispanic women with the Hp 2-2 phenotype. Vitamin C and E did not alter the risk of preeclampsia or late onset preeclampsia in Hispanic women with the Hp 1-1 or 2-1 phenotypes.

**Appendix S1**

In addition to the authors, *Eunice Kennedy Shriver* National Institute of Child Health and Human Development Maternal-Fetal Medicine Units Network members are as follows:

*University of Pittsburgh, Pittsburgh, Pennsylvania, United States:* J. Roberts, S. Caritis, T. Kamon (deceased), M. Cotroneo, D. Fischer

*University of Utah, Salt Lake City, Utah, United States:* M. Varner, P. Reed, K. Hill, and R. Silver (University Hospital), S. Quinn and F. Porter (LDS Hospital), V. Morby (McKay-Dee Hospital), J. Miller (Utah Valley Regional Medical Center)

*University of Alabama at Birmingham, Birmingham, Alabama, United States:* John C. Hauth, D.J. Rouse, A. Northen, P. Files, J. Grant, M. Wallace, K. Bailey

*Columbia University, New York, New York, United States:* R. Wapner, S. Bousleiman*,* R. Alcon, K. Saravia, F. Loffredo, A. Bayless (Christiana), C. Perez (St. Peter's University Hospital), M. Lake (St. Peter's University Hospital), M. Talucci

*University of North Carolina at Chapel Hill, Chapel Hill, North Carolina, United States:* K. Boggess, K. Dorman, J. Mitchell, K. Clark, S. Timlin

*Case Western Reserve University-MetroHealth Medical* Center*, Cleveland, Ohio, United States:* J. Bailit, C. Milluzzi, W. Dalton, C. Brezine, D. Bazzo

*University of Texas Southwestern Medical Center, Dallas, Texas, United States:* J. Sheffield, L. Moseley, M. Santillan, K. Buentipo, J. Price, L. S. Hermann, C. Melton, Y. Gloria-McCutchen, B. Espino

*Northwestern University, Chicago, Illinois, United States:* M. Dinsmoor (NorthShore University HealthSystem), T. Matson-Manning, G. Mallett

*University of Texas Health Science Center at Houston-Children’s Memorial Hermann Hospital, Houston, Texas, United States:* S. Blackwell, K. Cannon, S. Lege-Humbert, Z. Spears

*Brown University, Providence, Rhode Island, United States:* J. Tillinghast, M. Seebeck

*The Ohio State University, Columbus, Ohio, United States:* J. Iams, F. Johnson, S. Fyffe, C. Latimer, S. Frantz, S. Wylie

*Drexel University, Philadelphia, Pennsylvania, United States:* M. Talucci, M. Hoffman (Christiana), J. Benson (Christiana), Z. Reid, C. Tocci

*Wake Forest University Health Sciences, Winston-Salem, North Carolina, United States:* P. Meis, M. Swain

*Oregon Health & Science University, Portland, Oregon, United States:* W. Smith, L. Davis, E. Lairson, S. Butcher, S. Maxwell, D. Fisher

*University of Texas Medical Branch, Galveston, Texas, United States:* J. Moss, B. Stratton, G. Hankins, J. Brandon, C. Nelson-Becker, G. Olson, L. Pacheco

*Wayne State University, Detroit, Michigan, United States:* G. Norman, S. Blackwell, P. Lockhart, D. Driscoll, M. Dombrowski

*The George Washington University Biostatistics Center, Washington, DC, United States:* E. Thom, R. Clifton, T. Boekhoudt, L. Leuchtenburg

*National Heart, Lung, and Blood Institute, Bethesda, Maryland, United States:* V. Pemberton, J. Cutler, W. Barouch

*Eunice Kennedy Shriver National Institute of Child Health and Human Development, Bethesda, Maryland, United States*: S. Tolivaisa

*MFMU Steering Committee Chair (University of Texas Medical Center, Galveston, Texas, United States*): G.D. Anderson, M.D.
