# Supplementary material for: Haptoglobin Phenotype, Preeclampsia Risk and the Efficacy of Vitamin C and E Supplementation to Prevent Preeclampsia in a Racially Diverse Population
Source: PLoS One. 2013 Apr 3;8(4):e60479. doi: 10.1371/journal.pone.0060479 (PMC3616124; doi:10.1371/journal.pone.0060479)
Supplement: Table S1 — Prediction cohort subject characteristics. Values are mean ± SD or n (% within phenotype). Significant difference (p<0.05) from: *Hp 1-1, †Hp 2-1, ‡Hp 2-2. (DOC) [file pone.0060479.s002.doc]

**Table S1:** Prediction cohort subject characteristics

| **Subject Characteristics** | **Hp 1-1** (n=489) | **Hp 2-1** (n=1120) | **Hp 2-2** (n=725) | **Hp 2-1M** (n=55) | **p** |
| --- | --- | --- | --- | --- | --- |
| Age – years | 22.6 ± 4.5 | 23.4 ± 4.8* | 24.1 ± 4.9*^,†^ | 20.5 ± 3.4*^,†,‡^ | <0.01 |
| Gestational age at randomization – week | 11.4 ± 1.1 | 11.4 ± 1.1 | 11.5 ± 1.0 | 11.1 ± 1.1^‡^ | 0.01 |
| Race or ethnicity - n (% within phenotype) |  |  |  |  | <0.01 |
| White | 174 (36%) | 537 (48%) | 438 (60%) | 3 (5%) |  |
| Black | 161 (33%) | 256 (23%) | 124 (17%) | 47 (85%) |  |
| Hispanic | 148 (30%) | 306 (27%) | 145 (20%) | 4 (7%) |  |
| Other | 6 (1%) | 21 (2%) | 18 (2%) | 1 (2%) |  |
| Significance |  | * | *,† | ‡ |  |
| Pre-pregnancy body mass index - kg/m^2^ | 25.9 ± 6.7 | 25.3 ± 5.8 | 25.6 ± 6.2 | 27.3 ± 6.9 | 0.22 |
| Smoked during pregnancy - n (%) | 87 (18%) | 184 (16%) | 126 (17%) | 7 (13%) | 0.74 |
| Education - years | 12.6 ± 2.6 | 12.9 ± 2.6 | 13.3 ± 2.5*^,†^ | 12.3 ± 1.8^‡^ | <0.01 |
| Prenatal/multivitamin use prior to randomization - n (%) | 363 (74%) | 874 (78%) | 613 (85%)* | 47 (85%) | <0.01 |
| Previous pregnancy - n (%) | 102 (21%) | 249 (22%) | 176 (24%) | 15 (27%) | 0.43 |
| Family history of preeclampsia - n (%) | 70 (14%) | 150 (13%) | 93 (13%) | 6 (11%) | 0.84 |
| Blood pressure at entry (9-12 weeks) |  |  |  |  |  |
| Systolic - mmHg | 109 ± 10 | 110 ± 10 | 110 ± 10 | 111 ± 11 | 0.50 |
| Diastolic - mmHg | 65 ± 8 | 66 ± 8 | 67 ± 8 | 64 ± 9 | 0.05 |

Values are mean ± SD or n (% within phenotype).

Significant difference (p<0.05) from: *Hp 1-1, ^†^Hp 2-1, ^‡^Hp 2-2.
