# Supplementary material for: Haptoglobin Phenotype, Preeclampsia Risk and the Efficacy of Vitamin C and E Supplementation to Prevent Preeclampsia in a Racially Diverse Population
Source: PLoS One. 2013 Apr 3;8(4):e60479. doi: 10.1371/journal.pone.0060479 (PMC3616124; doi:10.1371/journal.pone.0060479)
Supplement: Table S2 — Outcomes according to Hp phenotype in the prediction cohort. *Adjusted for treatment group (vitamins vs. placebo), age, race/ethnicity, education, vitamin use prior to randomization, and diastolic blood pressure at randomization. †The primary outcome was severe PAH, or mild or severe PAH with elevated liver enzymes, elevated serum creatinine, thrombocytopenia, eclamptic seizure, fetal growth restriction, medically indicated preterm birth or perinatal death. (DOC) [file pone.0060479.s003.doc]

**Table S2:** Outcomes according to Hp phenotype in the prediction cohort

| **Outcome** | **n** | **Hp 1-1** (n=489) | **Hp 2-1** (n=1120) | **Hp 2-2** (n=725) | **Hp 2-1M** (n=55) | **p*** |
| --- | --- | --- | --- | --- | --- | --- |
| Primary Outcome^†^ | 159 | 42 (8.6%) | 68 (6.1%) | 49 (6.8%) | 4 (7.3%) | 0.37 |
| Gestational Hypertension | 609 | 126 (28.4%) | 277 (26.4%) | 183 (27.4%) | 23 (45.1%) | 0.84 |
| Preeclampsia | 175 | 45 (9.2%) | 71 (6.3%) | 55 (7.6%) | 4 (7.3%) | 0.19 |
| Severe Preeclampsia | 71 | 18 (3.9%) | 29 (2.7%) | 23 (3.3%) | 1 (1.9%) | 0.50 |
| Early Onset Preeclampsia | 32 | 9 (2.0%) | 14 (1.3%) | 9 (1.3%) | 0 (0%) | 0.69 |
| Late Onset Preeclampsia | 143 | 36 (7.5%) | 57 (5.1%) | 46 (6.4%) | 4 (7.3%) | 0.21 |

*Adjusted for treatment group (vitamins vs. placebo), age, race/ethnicity, education, vitamin use prior to randomization, and diastolic blood pressure at randomization.

^†^The primary outcome was severe PAH, or mild or severe PAH with elevated liver enzymes, elevated serum creatinine, thrombocytopenia, eclamptic seizure, fetal growth restriction, medically indicated preterm birth or perinatal death.
