# Supplementary material for: Haptoglobin Phenotype, Preeclampsia Risk and the Efficacy of Vitamin C and E Supplementation to Prevent Preeclampsia in a Racially Diverse Population
Source: PLoS One. 2013 Apr 3;8(4):e60479. doi: 10.1371/journal.pone.0060479 (PMC3616124; doi:10.1371/journal.pone.0060479)
Supplement: Table S3 — Subject Characteristics for Subjects not in the Prediction Cohort, and in the Weighted Case-Control Cohort. Values are mean ± SD or n (%). (DOC) [file pone.0060479.s004.doc]

**Table S3:** Subject Characteristics for Subjects not in the Prediction Cohort, and in the Weighted Case-Control Cohort

| **Subject Characteristics** | **Subjects not in the Prediction Cohort of the Original Study** | | **Weighted Case-Control Cohort** | |
| --- | --- | --- | --- | --- |
|  | **Placebo** (n=3,877) | **Vitamins** (n=3,882) | **Placebo** (n=3,977) | **Vitamins** (n=3,599) |
| Age – years | 23.5 ± 5.4 | 23.5 ± 5.3 | 23.4 ± 5.2 | 23.4 ± 5.2 |
| Gestational age at randomization – week | 14.0 ± 1.9 | 14.0 ± 2.0 | 13.9 ± 1.9 | 14.0 ± 1.9 |
| Race or ethnicity - n (%) |  |  |  |  |
| White | 1,531 (39%) | 1,554 (40%) | 1,610 (40%) | 1,409 (39%) |
| Black | 1,004 (26%) | 967 (25%) | 1,034 (26%) | 892 (25%) |
| Hispanic | 1,269 (33%) | 1,296 (33%) | 1,246 (31%) | 1,234 (34%) |
| Other | 73 (2%) | 65 (2%) | 88 (2%) | 63 (2%) |
| Pre-pregnancy body mass index - kg/m^2^ | 25.4 ± 5.9 | 25.3 ± 5.9 | 25.0 ± 5.4 | 24.8 ± 5.6 |
| Smoked during pregnancy - n (%) | 579 (15%) | 609 (16%) | 676 (17%) | 532 (15%) |
| Education - years | 12.7 ± 2.8 | 12.7 ± 2.7 | 12.6 ± 2.8 | 12.7 ± 2.8 |
| Vitamin use prior to randomization - n (%) | 2,938 (76%) | 2,955 (76%) | 3,002 (76%) | 2,703 (75%) |
| Previous pregnancy - n (%) | 902 (23%) | 885 (23%) | 965 (24%) | 862 (24%) |
| Family history of preeclampsia - n (%) | 493 (13%) | 511 (13%) | 545 (14%) | 449 (12%) |
| Blood pressure at entry (9-12 weeks) |  |  |  |  |
| Systolic - mmHg | 109 ± 10 | 109 ± 10 | 108 ± 10 | 107 ± 10 |
| Diastolic - mmHg | 65 ± 8 | 65 ± 8 | 64 ± 8 | 64 ± 8 |

Values are mean ± SD or n (%).
