# Supplementary material for: Haptoglobin Phenotype, Preeclampsia Risk and the Efficacy of Vitamin C and E Supplementation to Prevent Preeclampsia in a Racially Diverse Population
Source: PLoS One. 2013 Apr 3;8(4):e60479. doi: 10.1371/journal.pone.0060479 (PMC3616124; doi:10.1371/journal.pone.0060479)
Supplement: Table S4 — Case-control cohort weighted subject characteristics. Values are mean ± SD or n (%). Abbreviations: NA; not applicable. Statistical comparisons for race/ethnicity were not performed, as cases and controls were matched for race/ethnicity. Significant difference (p<0.05) from: *Hp 1-1, †Hp 2-1, ‡Hp 2-2. (DOC) [file pone.0060479.s005.doc]

**Table S4:** Case-control cohort weighted subject characteristics

| **Subject Characteristics** | **Hp 1-1** (n=1713 ) | **Hp 2-1** (n=3721 ) | **Hp 2-2** (n=2034) | **Hp 2-1M** (n=106) | **p** |
| --- | --- | --- | --- | --- | --- |
| Age – years | 22.3 ± 5.2 | 23.6 ± 5.2* | 23.9 ± 5.0* | 20.0 ± 3.7*^,†.‡^ | <0.01 |
| Gestational age at randomization – week | 13.9 ± 2.0 | 13.9 ± 1.9 | 14.1 ± 1.8 | 14.0 ± 2.3 | 0.56 |
| Race or ethnicity - n (% within phenotype) |  |  |  |  | NA |
| White | 444 (26%) | 1605 (43%) | 970 (48%) | 0 (0%) |  |
| Black | 618 (36%) | 803 (22%) | 418 (21%) | 85 (81%) |  |
| Hispanic | 631 (37%) | 1249 (34%) | 579 (28%) | 21 (19%) |  |
| Other | 19 (1%) | 63 (2%) | 68 (3%) | 0 (0%) |  |
| Pre-pregnancy body mass index - kg/m^2^ | 25.1 ± 6.1 | 24.8 ± 5.3 | 24.8 ± 5.2 | 26.3 ± 9.3 | 0.56 |
| Smoked during pregnancy - % | 265 (15%) | 537 (14%) | 389 (19%) | 16 (16%) | 0.14 |
| Education - years | 12.0 ± 3.1 | 12.7 ± 2.8* | 13.1 ± 2.5*^,†^ | 11.8 ± 2.0^‡^ | <0.01 |
| Prenatal/multivitamin use prior to randomization - n (%) | 1154 (67%) | 2885 (78%)* | 1581 (78%)* | 85 (81%) | <0.01 |
| Previous pregnancy - n (%) | 418 (24%) | 835 (22%) | 555 (27%) | 18 (17%) | 0.20 |
| Family history of preeclampsia - n (%) | 249 (15%) | 484 (13%) | 248 (12%) | 12 (12%) | 0.75 |
| Blood pressure at entry (9-12 weeks) |  |  |  |  |  |
| Systolic - mmHg | 108 ± 11 | 108 ± 10 | 107 ± 10 | 109 ± 9 | 0.18 |
| Diastolic - mmHg | 65 ± 9 | 64 ± 7 | 65 ± 7 | 64 ± 8 | 0.69 |

Values are mean ± SD or n (%). Abbreviations: NA; not applicable. Statistical comparisons for race/ethnicity were not performed, as cases and controls were matched for race/ethnicity.

Significant difference (p<0.05) from: *Hp 1-1, ^†^Hp 2-1, ^‡^Hp 2-2.
