# Supplementary material for: Haptoglobin Phenotype, Preeclampsia Risk and the Efficacy of Vitamin C and E Supplementation to Prevent Preeclampsia in a Racially Diverse Population
Source: PLoS One. 2013 Apr 3;8(4):e60479. doi: 10.1371/journal.pone.0060479 (PMC3616124; doi:10.1371/journal.pone.0060479)
Supplement: Table S5 — Outcomes according to Hp phenotype and race/ethnicity in the case-control cohort. Hp 1-1 was the reference group for odds ratios. Hp phenotype was determined in 769 black women, 579 Hispanic women, and 716 women of white or other race. Each observation was weighted to reflect the pregnancy outcomes and racial distribution of the full cohort, excluding women in the prediction study (3,170 white/other women, 1,839 black women, 2,459 Hispanic women). Abbreviations: OR, odds ratio; CI, confidence interval. *Adjusted for group (treatment vs. placebo), race/ethnicity, age, education, vitamin use and diastolic blood pressure at randomization †Significantly different from 1, p<0.05. (DOC) [file pone.0060479.s006.doc]

**Table S5:** Outcomes according to Hp phenotype and race/ethnicity in the case-control cohort

| **Outcome** | **Cases**  **(n)** | **Hp 2-1*** **OR (95% CI)** | **Hp 2-2*** **OR (95% CI)** |
| --- | --- | --- | --- |
| White/Other |  |  |  |
| Primary Outcome | 150 | 0.83 (0.48, 1.42) | 0.79 (0.45, 1.41) |
| Preeclampsia | 159 | 1.19 (0.66, 2.16) | 1.42 (0.77, 2.62) |
| Severe Preeclampsia | 64 | 1.91 (0.75, 4.92) | 1.75 (0.66, 4.63) |
| Early Onset Preeclampsia | 45 | 1.66 (0.52, 5.28) | 2.47 (0.78, 7.79) |
| Late Onset Preeclampsia | 114 | 1.09 (0.57, 2.09) | 1.17 (0.59, 2.31) |
| Black |  |  |  |
| Primary Outcome | 176 | 1.64 (1.08, 2.48)^†^ | 1.27 (0.77, 2.09) |
| Preeclampsia | 180 | 1.26 (0.84, 1.89) | 1.04 (0.64, 1.70) |
| Severe Preeclampsia | 89 | 1.38 (0.81, 2.36) | 0.93 (0.45, 1.83) |
| Early Onset Preeclampsia | 64 | 1.21 (0.64, 2.27) | 1.09 (0.52, 2.28) |
| Late Onset Preeclampsia | 116 | 1.29 (0.79, 2.09) | 1.01 (0.56, 1.84) |
| Hispanic |  |  |  |
| Primary Outcome | 89 | 0.83 (0.47, 1.46) | 0.82 (0.40, 1.67) |
| Preeclampsia | 165 | 0.70 (0.45, 1.09) | 0.56 (0.31, 1.00)^†^ |
| Severe Preeclampsia | 58 | 0.63 (0.32, 1.22) | 0.47 (0.19, 1.16) |
| Early Onset Preeclampsia | 33 | 0.34 (0.14, 0.82)^†^ | 0.39 (0.13, 1.22) |
| Late Onset Preeclampsia | 132 | 0.84 (0.51, 1.37) | 0.62 (0.32, 1.19) |

Hp 1-1 was the reference group for odds ratios. Hp phenotype was determined in 769 black women, 579 Hispanic women, and 716 women of white or other race. Each observation was weighted to reflect the pregnancy outcomes and racial distribution of the full cohort, excluding women in the prediction study (3,170 white/other women, 1,839 black women, 2,459 Hispanic women).

Abbreviations: OR, odds ratio; CI, confidence interval.

*Adjusted for group (treatment vs. placebo), race/ethnicity, age, education, vitamin use and diastolic blood pressure at randomization

^†^Significantly different from 1, p<0.05.
