# Supplementary material for: Haptoglobin Phenotype, Preeclampsia Risk and the Efficacy of Vitamin C and E Supplementation to Prevent Preeclampsia in a Racially Diverse Population
Source: PLoS One. 2013 Apr 3;8(4):e60479. doi: 10.1371/journal.pone.0060479 (PMC3616124; doi:10.1371/journal.pone.0060479)
Supplement: Table S6 — Interaction between Hp phenotype and treatment in case-control cohort. *Adjusted for vitamin use, age, education and diastolic blood pressure at randomization (DOC) [file pone.0060479.s007.doc]

**Table S6:** Interaction between Hp phenotype and treatment in case-control cohort

| **Outcome** | **Hp Phenotype x Treatment p value*** | | | |
| --- | --- | --- | --- | --- |
|  | **All** | **White/Other** | **Black** | **Hispanic** |
| Primary Outcome | 0.92 | 0.78 | 0.91 | 0.26 |
| Preeclampsia | 0.10 | 0.51 | 0.79 | 0.02 |
| Severe Preeclampsia | 0.92 | 0.57 | 0.71 | 0.11 |
| Early Onset Preeclampsia | 0.93 | 0.91 | 0.99 | 0.38 |
| Late Onset Preeclampsia | 0.05 | 0.39 | 0.73 | 0.02 |

*Adjusted for vitamin use, age, education and diastolic blood pressure at randomization
