# Supplementary material for: Haptoglobin Phenotype, Preeclampsia Risk and the Efficacy of Vitamin C and E Supplementation to Prevent Preeclampsia in a Racially Diverse Population
Source: PLoS One. 2013 Apr 3;8(4):e60479. doi: 10.1371/journal.pone.0060479 (PMC3616124; doi:10.1371/journal.pone.0060479)
Supplement: Table S7 — Odds ratios for the effect of treatment in Hispanic women in the case-control cohort. *Adjusted for vitamin use, age, education and diastolic blood pressure at randomization (DOC) [file pone.0060479.s008.doc]

**Table S7:** Odds ratios for the effect of treatment in Hispanic women in the case-control cohort

| **Outcome** | **Phenotype** | **OR (95% CI)*** | **p** |
| --- | --- | --- | --- |
| Preeclampsia | Hp 1-1 | 0.70 (0.36, 1.37) | 0.30 |
|  | Hp 2-1 | 1.21 (0.71, 2.07) | 0.48 |
|  | Hp 2-2 | 4.08 (1.59, 10.43) | <0.01 |
| Late Onset Preeclampsia | Hp 1-1 | 0.68 (0.32, 1.43) | 0.31 |
|  | Hp 2-1 | 1.31 (0.74, 2.32) | 0.35 |
|  | Hp 2-2 | 4.37 (1.54, 12.43) | <0.01 |

*Adjusted for vitamin use, age, education and diastolic blood pressure at randomization
