# Supplementary material for: Haptoglobin Phenotype, Preeclampsia Risk and the Efficacy of Vitamin C and E Supplementation to Prevent Preeclampsia in a Racially Diverse Population
Source: PLoS One. 2013 Apr 3;8(4):e60479. doi: 10.1371/journal.pone.0060479 (PMC3616124; doi:10.1371/journal.pone.0060479)
Supplement: Table S8 — Subject Characteristics for the Original Study and the Weighted Pooled Cohort Values are mean ± SD or n (%). *Values for the 9,969 women with known pregnancy outcomes in the original study. Women who were lost to follow-up (n = 183) were excluded. Weights for this analysis were based on race/ethnicity and pregnancy outcome, therefore these women were not eligible to be selected for the pooled cohort analysis. (DOC) [file pone.0060479.s009.doc]

**Table S8:** Subject Characteristics for the Original Study and the Weighted Pooled Cohort

| **Subject Characteristics** | **All Subjects in the Original Study*** | | **Weighted Pooled Cohort** | |
| --- | --- | --- | --- | --- |
|  | **Placebo** (n=4,976) | **Vitamins** (n=4,993) | **Placebo** (n=4,800) | **Vitamins** (n=5,163) |
| Age – years | 23.5 ± 5.3 | 23.5 ± 5.2 | 23.4 ± 5.1 | 23.4 ± 5.1 |
| Gestational age at randomization – week | 13.4 ± 2.1 | 13.4 ± 2.1 | 13.3 ± 2.0 | 13.4 ± 2.1 |
| Race or ethnicity - n (%) |  |  |  |  |
| White | 2,080 (41.8%) | 2,106 (42.2%) | 2,184 (42%) | 1,987 (41%) |
| Black | 1,273 (25.6%) | 1,245 (24.9%) | 1,323 (26%) | 1,189 (25%) |
| Hispanic | 1,524 (30.6%) | 1,559 (31.2%) | 1,543 (30%) | 1,540 (32%) |
| Other | 99 (2.0%) | 83 (1.8%) | 114 (2%) | 83 (2%) |
| Pre-pregnancy body mass index - kg/m^2^ | 25.4 ± 5.9 | 25.4 ± 6.0 | 25.1 ± 5.6 | 25.0 ± 5.8 |
| Smoked during pregnancy - n (%) | 763 (15%) | 788 (16%) | 878 (17%) | 734 (15%) |
| Education - years | 12.8 ± 2.7 | 12.8 ± 2.7 | 12.7 ± 2.7 | 12.7 ± 2.8 |
| Vitamin use prior to randomization - n (%) | 3,838 (77%) | 3,853 (77%) | 3,952 (77%) | 3,649 (76%) |
| Previous pregnancy - n (%) | 1,149 (23%) | 1,138 (23%) | 1,232 (24%) | 1,136 (24%) |
| Family history of preeclampsia - n (%) | 659 (13%) | 638 (13%) | 708 (14%) | 604 (13%) |
| Blood pressure at entry (9-12 weeks) |  |  |  |  |
| Systolic - mmHg | 109 ± 10 | 109 ± 10 | 108 ± 10 | 108 ± 10 |
| Diastolic - mmHg | 65 ± 8 | 66 ± 8 | 65 ± 8 | 65 ± 8 |

Values are mean ± SD or n (%).

*Values for the 9,969 women with known pregnancy outcomes in the original study. Women who were lost to follow-up (n=183) were excluded. Weights for this analysis were based on race/ethnicity and pregnancy outcome, therefore these women were not eligible to be selected for the pooled cohort analysis.
