# Supplementary material for: Haptoglobin Phenotype, Preeclampsia Risk and the Efficacy of Vitamin C and E Supplementation to Prevent Preeclampsia in a Racially Diverse Population
Source: PLoS One. 2013 Apr 3;8(4):e60479. doi: 10.1371/journal.pone.0060479 (PMC3616124; doi:10.1371/journal.pone.0060479)
Supplement: Table S9 — Hp phenotype prevalence by race in the weighted pooled cohort Values are n (% within race). (DOC) [file pone.0060479.s010.doc]

**Table S9:** Hp phenotype prevalence by race in the weighted pooled cohort

| **Race or Ethnicity** | **Hp 1-1** (n=2,201) | **Hp 2-1** (n=4,840) | **Hp 2-2** (n=2,760) | **Hp 2-1M** (n=146) |
| --- | --- | --- | --- | --- |
| White | 618 (14.8%) | 2,142 (51.4%) | 1,408 (33.8%) | 3 (0.1%) |
| Black | 779 (31.0%) | 1,059 (42.2%) | 542 (21.6%) | 132 (5.3%) |
| Hispanic | 779 (25.4%) | 1,555 (50.7%) | 724 (23.6%) | 10 (0.3%) |
| Other | 25 (12.8%) | 84 (42.9%) | 86 (43.9%) | 1 (0.5%) |

Values are n (% within race).
