# Supplementary material for: Haptoglobin Phenotype, Preeclampsia Risk and the Efficacy of Vitamin C and E Supplementation to Prevent Preeclampsia in a Racially Diverse Population
Source: PLoS One. 2013 Apr 3;8(4):e60479. doi: 10.1371/journal.pone.0060479 (PMC3616124; doi:10.1371/journal.pone.0060479)
Supplement: Table S10 — Subject Characteristics for Hispanic vs. non-Hispanic Women in the Weighted Pooled Cohort. Values are mean ± SD unless otherwise indicated. (DOC) [file pone.0060479.s011.doc]

**Table S10:** Subject Characteristics for Hispanic vs. non-Hispanic Women in the Weighted Pooled Cohort

| **Subject Characteristics** | **Hispanic** (n=1,188) | **Non-Hispanic** (n=3,306) | **p** |
| --- | --- | --- | --- |
| Age – years | 22.3 ± 4.5 | 23.5 ± 5.1 | <0.01 |
| Gestational age at randomization – week | 12.6 ± 2.0 | 12.6 ± 2.0 | 0.65 |
| Race or ethnicity - n (%) |  |  | NA |
| White | 0 (0%) | 1,840 (55.7%) |  |
| Black | 0 (0%) | 1,392 (42.1%) |  |
| Hispanic | 1,188 (100%) | 0 (0%) |  |
| Other | 0 (0%) | 74 (2.2%) |  |
| Pre-pregnancy body mass index - kg/m^2^ | 24.9 ± 4.9 | 26.0 ± 6.7 | <0.01 |
| Smoked during pregnancy - n (%) | 64 (5.4%) | 704 (21.3%) | <0.01 |
| Education - years | 10.8 ± 2.8 | 13.4 ± 2.1 | <0.01 |
| Vitamin use prior to randomization - n (%) | 511 (43.0%) | 3,011 (91.1%) | <0.01 |
| Previous pregnancy - n (%) | 243 (20.5%) | 812 (24.6%) | <0.01 |
| Family history of preeclampsia - n (%) | 202 (17.0%) | 430 (13.0%) | <0.01 |
| Blood pressure at entry (9-12 weeks) |  |  |  |
| Systolic - mmHg | 106 ± 10 | 111 ± 10 | <0.01 |
| Diastolic - mmHg | 64 ± 7 | 66 ± 8 | <0.01 |

Values are mean ± SD unless otherwise indicated.
